# Protein induced by vitamin K absence or antagonist-II production is a strong predictive marker for extrahepatic metastases in early hepatocellular carcinoma: a prospective evaluation

**DOI:** 10.1186/1471-2407-11-435

**Published:** 2011-10-10

**Authors:** Hyun-Mi Bae, Jeong-Hoon Lee, Jung-Hwan Yoon, Yoon Jun Kim, Dae Seog Heo, Hyo-Suk Lee

**Affiliations:** 1 Department of Internal Medicine, Seoul National University College of Medicine, Seoul, South Korea; 2 Division of Oncology, Department of Internal Medicine, Incheon St. Mary's Hospital, Catholic University, Incheon, Korea; 3 Liver Research Institute, Seoul National University College of Medicine, Seoul, South Korea; 4 Cancer Research Institute, Seoul National University College of Medicine, Seoul, South Korea

**Keywords:** Protein induced by vitamin K absence or antagonist-II, hepatocellular carcinoma, metastases, predictive marker

## Abstract

**Background:**

Clinicians often experience extrahepatic metastases associated with hepatocellular carcinoma (HCC), even if no evidence of intrahepatic recurrence after treatment is observed. We investigated the pretreatment predictors of extrahepatic metastases in HCC patients.

**Methods:**

Patients diagnosed with HCC without evidence of extrahepatic metastases were prospectively enrolled. We evaluated the correlation between extrahepatic metastases and pretreatment clinical variables, including serum tumor markers.

**Results:**

A total of 354 patients were included. Seventy-six patients (21%) had extrahepatic metastases during the observation period (median, 25.3 months; range, 0.6-51.3 months). Cox regression multivariate analysis showed that serum protein induced by vitamin K absence or antagonist-II (PIVKA-II) production levels, the intrahepatic tumor stage, platelet count, and portal vein thrombosis were independent risk factors for extrahepatic metastases. Patients with a PIVKA-II production ≥ 300 mAU/mL had a 2.7-fold (95% confidence interval; 1.5-4.8; P < 0.001) and 3.7-fold (95% confidence interval; 2.0-6.6; P < 0.001) increased risk for extrahepatic metastases after adjustment for stage, platelet count, alpha-fetoprotein ≥ 400 ng/mL, and portal vein thrombosis according to the AJCC and BCLC staging systems, respectively.

**Conclusion:**

PIVKA-II production levels might be a good candidate predictive marker for extrahepatic HCC metastases, especially in patients with smaller and/or fewer tumors in the liver with in stages regardless of serum alpha-fetoprotein.

## Background

Hepatic resection and liver transplantation are an established curative treatment for early stage HCC patients [1-3]. However, few good candidates are available for surgical resection and liver transplantation due to surgical risks associated with liver cirrhosis and the limited number of available donors, respectively. On the other hand, alternative therapeutic approaches, such as percutaneous ethanol injection (PEI) or radiofrequency ablation (RFA) for small HCCs [3,4] and transarterial chemoembolization (TACE) for multinodular hypervascular HCC, have been widely applied for the treatment of patients with HCC [5,6]. Although an increased number of therapeutic options may improve treatment results for small HCCs, a significant proportion of patients that undergo these procedures have intrahepatic *de novo *recurrence or metastases [7,8]. It was reported that the percentages of intrahepatic metastases and multicentric occurrences found after initial treatment of small hepatocellular carcinomas less than 2 cm in diameter was 23.7% after 1 year, 64.5% after 3 years, and 76.1% after 5 years [8].

Sometimes, clinicians experience extrahepatic metastases even if a primary lesion is not found after surgical resection or liver transplantation. In addition, extrahepatic metastases may occur after locoregional therapies for early stage HCC and a long-term remission. As recent advances have been made in our understanding of the molecular pathways of HCC, the metastatic potential of early HCC has been confirmed by gene expression assessment using microarray analysis [9]. In addition, an oral multikinase inhibitor that targets serine/threonine and tyrosine kinase receptors has been shown to result in decreased tumor growth and inhibition of angiogenesis in patients with HCC [10-12]. If we could determine the patients at increased risk for extrahepatic metastases at an early timepoint with specific predictive factors, we could select good candidates for surgical treatment and prevent unnecessary liver transplantation or surgical resection. Furthermore, agents targeted to the appropriate tumors at the appropriate time could control the development of distant metastases. However, the factors that predict metastases, including specific tumor markers, are not yet identified for patients with HCC.

In our previous pilot study of patients who were followed after treatment of HCC, those with high serum levels of protein induced by vitamin K absence or antagonist-II production (PIVKA-II) during follow up showed an increased rate of development of metastases at an early stage, even if they had well-controlled primary liver lesions without portal vein thromboses. In this study, we prospectively investigated the pretreatment predictors of extrahepatic metastases in HCC patients.

## Methods

### Patients

Four hundred seventy-six consecutive patients who were diagnosed with HCC between September 2005 and July 2008 at Seoul National University Hospital in Seoul, Korea, were included. Patients whose serum levels of PIVKA-II and AFP at the time of diagnosis were not available (64 patients), those who had metastases at the time of diagnosis (43), those who were untreated (15), were excluded. Any patients who may have been taking warfarin would have also been excluded, though none of the patients in this study were treated with it. Accordingly, 354 patients were enrolled into this prospective cohort study, which was approved by the Institutional Review Board of Seoul National University Hospital. We obtained written informed consents from all patients enrolled.

### Diagnosis

A definitive diagnosis of HCC was confirmed based on typical hypervascular radiologic features and serum alpha-fetoprotein (AFP) levels or histologic findings by AASLD guidelines. All patients underwent testing for liver function, hepatitis B and C profiles, and serum AFP and PIVKA-II levels; a chest PA and dynamic CT of the abdomen and pelvis were obtained. If nodules were noted on the chest PA, a chest CT or biopsy were performed to exclude lung metastases. Patients complaining of bone pain had a bone scan. Follow-up after the initial treatment consisted of blood tests, monitoring of tumor markers, chest PA, and a dynamic CT including the pelvis once or twice within 6 months.

### AFP and PIVKA-II determinations

The serum concentration of PIVKA-II was assayed by an enzyme-linked immunoassay kit using Eitest (cut-off value, 40 mAU/mL; Sanko Junyaku Co., Tokyo, Japan). The AFP was measured using the AFP IRMA system (cut-off value, 20 ng/mL; Immunotech, Beckman Coulter, Fullerton, CA, USA). Both the serum AFP and PIVKA-II were measured when patients were initially diagnosed with HCC.

### Variables

The following variables, measured at the time of diagnosis, were included in the analysis to identify predictors of extrahepatic metastases: age; gender; tumor stage; tumor size; Child-Pugh class; serum levels of AFP, PIVKA-II, albumin, and total bilirubin; and prothombin time (PT). We analyzed two different tumor stage systems (the American Joint Committee on Cancer [AJCC] and the Barcelona Clinic Liver Cancer [BCLC] staging systems) to evaluate the correlation between extrahepatic metastases and other clinical variables [13,14]. Tumor markers were used with both continuous and dichotomized variables with cut-off values for AFP at ≥ 400 ng/mL and PIVKA-II at ≥ 300 mAU/mL when we analyzed the predictive factors for extrahepatic metastases, other clinical factors, and tumor markers.

### Locoregional treatment for HCC

Patients were treated by PEI or RFA for lesions < 4 cm in diameter. Hepatic resection was carried out if the tumors were small and the patients had good liver function without significant co-morbidities. TACE was performed in patients with multiple hypervascular intrahepatic tumors.

### Statistics analysis

We evaluated the correlation between extrahepatic metastases, serum levels of tumor markers (AFP and PIVKA-II), and clinical findings according to the AJCC and BCLC staging systems. Univariate analyses to identify predictors of extrahepatic metastases were performed using the Kaplan-Meier method or Cox-regression and the variables at the time of diagnosis. The differences between groups were tested for significance using the log-rank test. The variables which were significantly associated with extrahepatic metastases in the univariate analyses were included in the multivariate analysis with the Cox proportional hazards regression model using a forward stepwise approach to identify independent predictors of extrahepatic metastases. All statistical analyses were performed using SPSS 12.0 (SPSS, Inc., Chicago, IL, USA). For all tests, a *P*-value < 0.05 was considered statistically significant.

## Results

### Baseline characteristics

Two hundred sixty-five among 354 patients (75%) were males, and the median age was 56 years (range, 19-82 years). The median duration of follow-up of all patients was 25.3 months (range, 0.6-51.3 months). The baseline characteristics of the study population for all patients are shown in Table 1. The percentages of patients who were seropositive for hepatitis B virus surface antigen and anti-hepatitis C virus antibody were 95.2% and 3.7%, respectively. The percentages of patients in Child-Pugh classes A, B, and C were 69.8%, 26.0%, and 4.2%, respectively. At the time of diagnosis, the proportion of patients with an AFP ≥ 400 ng/mL was 31.1%, and 28% of patients had a PIVKA-II ≥ 300 mAU/mL. The median values for platelet count and PT (%) were 121, 000/mm^3 ^and 83%, respectively; the median values for albumin and bilirubin were 3.7 g/dL and 1.0 mg/dL, respectively. Of 354 patients, 76 (21%) had extrahepatic metastases during the observation period. The most common places for metastases to occur are the lungs (57%), bones (20%), metastatic lymph nodes (36%), adrenal gland (7%), peritoneal seeding (5%), spleen (3%), and the brain (3%).

**Table 1:** Baseline characteristics

Characteristics		n = 354
Age (years)^†^		56 (19-82)

Sex	Male	265 (74.9%)

Etiology of chronic liver disease	Hepatitis B virus	337 (95.2%)
	Hepatitis C virus	13 (3.7%)
Tumor size	Non-B, non-C < 5 cm	4 (1.1%) 261 (73.7%)
	≥ 5 cm	93 (26.2%)
Tumor number	1	216 (61.0%)
	2-3	57 (16.1%)
	> 3	81 (22.8%)

Treatment modality	TACE	204 (57.6%)
	PEIT	74 (20.9%)
	RFA	42 (11.9%)
	Surgery	34 (9.6%)

BCLC Tumor stage	A: Early	224 (63.1%)
	B: Intermediate	48 (13.5%)
	C: advanced	67 (18.9%)
	D: end-stage	15 (4.2%)

AJCC Tumor stage	I	170 (48.0%)
	II	86 (24.6%)
	III	98 (27.4%)

Portal vein thrombosis	Present	65 (18.4%)

Child-Pugh class	A	247 (69.8%)
	B	92 (26.0%)
	C	15 (4.2%)

Serum AFP		52.0 (2.2-3, 560, 000)
(ng/mL)	< 400	244 (68.9%)
	≥ 400	110 (31.1%)

Serum PIVKA-II		54.0 (2.0-42, 780)
(mAU/mL)	< 300	255 (72.0%)
	≥ 300	99 (28.0%)

Laboratory parameters	Platelet count (10^3^/μL)^†^	121 (18-610)

	PT (%)^†^	83 (36-126)

	AST (IU/L)^†^	50 (17-2036)

	ALT (IU/L)^†^	43 (6-1775)

	Albumin (g/dL)^†^	3.7 (2.1-5.0)

	Total bilirubin (mg/dL)^†^	1.0 (0.3-31.8)

Values in the column are represented as number of patients (%) or median value (range).

Abbreviation: TACE, transarterial chemoembolization; RFA, radiofrequency ablation; PEI, percutaneous ethanol injection; AFP, alpha-fetoprotein; PIVKA-II, Protein induced by vitamin K absence or antagonist-II; PT, prothrombin time; AST, aspartate aminotransaminase; ALT, alanine aminotransferase.

† all were used with continuous variables.

### Predictive factors for extrahepatic metastases

#### All patients

**Figure 1:**
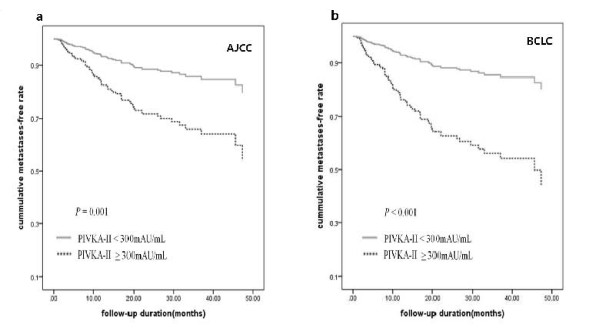
**The median time to extrahepatic metastases in patients with a PIVKA-II ≥ 300 mAU/mL**. The cumulative metastasis-free rate was analyzed in a multivariate Cox regression model in patients with a PIVKA-II ≥ 300 in AJCC (1a; Hazard Ratio, HR = 2.7, 95% confidence interval [CI] = 1.535-4.812, adjusted P = 0.001) and BCLC stage BCLC (1b: HR = 3.7, 95% CI = 2.073-6.560, adjusted P < 0.001) adjusting for such covariates as platelet count, AFP ≥ 400, portal vein thrombosis, and stage.

Of the entire cohort of patients, the AJCC (P < 0.001) and BCLC tumor stage (P < 0.001), serum PIVKA-II level (P < 0.001), AFP level (P = 0.008), portal vein thrombosis (P < 0.001), and platelet count (P = 0.0004) were significantly associated with the presence of extrahepatic metastases based on univariate analyses (Table 2). However, age, bilirubin and albumin levels, and prothorombin time (PT) failed to show a significant association with extrahepatic metastases. When we applied the AJCC and BCLC staging systems in the subsequent Cox-regression multivariate analysis, PIVKA-II levels with continuous variables (P = 0.001 and P < 0.001), portal vein thrombosis (P = 0.126 and P < 0.001), tumor stage (P < 0.001 and P < 0.001), and platelet count (P = 0.019 and P = 0.011) were shown to be independent risk factors for extrahepatic metastases, respectively. We performed multivariate analysis with stage, portal vein thrombosis, platelet count, and tumor markers according to cut-off values of tumor markers for AFP (≥ 400 ng/mL) and PIVKA-II (≥ 300 mAU/mL). It was consistently shown that PIVKA-II ≥ 300 mAU/mL, portal vein thrombosis (in BCLC) and platelet count were independent risk factors for extrahepatic metastases based on multivariate analysis (Table 3). However, AFP did not significantly increase the hazard ratio of extrahepatic metastasis in any combination of tumor markers based on multivariate analysis. The patients with a PIVKA-II ≥ 300 mAU/mL had a 2.7-fold (95% confidence interval [CI], 1.535-4.812, P = 0.001) and a 3.7-fold (95% confidence interval [CI], 2.073-6.560, P < 0.001) in AJCC and BCLC hazard ratios of extrahepatic metastases, respectively, when the those with AFP ≥ 400 and PIVKA-II ≥ 300 in Cox regression analysis after adjustment for portal vein thrombosis, platelet count, and stage were applied. Figure 1 shows the time to extrahepatic metastases based on Cox regression analysis in patients with a PIVKA-II ≥ 300 in AJCC and BCLC stages after adjustment for portal vein thrombosis, AFP ≥ 400 platelet count, and stage.

**Table 2:** Univariate analysis in overall patients

Characteristics	Hazard ratio (95% CI)	P value*
Age (years)^†^	0.981 (0.958-1.004)	0.106

Sex	0.937 (0.558-1.576)	0.807

Etiology of CLD	0.361 (0.090-1.439)	0.149

Portal vein thrombosis	6.716 (4.169-10.819)	< 0.001

Child-Pugh class	1.264 (0.833-1.918)	0.270

AJCC Tumor stage	4.759 (3.410-6.643)	< 0.001

BCLC Tumor stage	1.532 (1.383-1.698)	< 0.001

Serum AFP^†^	1.0000007 (1.0000002-1.0000013)	0.008
Serum AFP (≥ 400 ng/mL)	3.666 (2.332-5.762)	< 0.001

Serum PIVKA-II^†^	1.000102 (1.00007-1.00012)	< 0.001
Serum PIVKA-II (≥ 300 mAU/mL)	8.701 (5.394-14.035)	< 0.001

Platelet count (≤ 130 × 10^3^/μL)	2.319 (1.454-3.698)	0.0004

PT (≤ 80%)	0.737 (0.460-1.179)	0.203

Albumin (≥ 3.5 g/dl)	0.790 (0.498-1.252)	0.316

Total bilirubin (≥ 1.5 mg/dL)	1.174 (0.287-4.796)	0.823

Values in the column are represented as number of patients (%) or median value (range).

Abbreviation: CLD, chronic liver disease; AFP, alpha-fetoprotein; PIVKA-II, Protein induced by vitamin K absence or antagonist-II; PT, prothrombin time; AST, aspartate aminotransaminase; ALT, alanine aminotransferase.

*P-values for each clinical variable calculated using Cox regression analysis.

†all were used with continuous variables.

**Table 3:** Multivariate analysis in overall patients according to AJCC or BCLC tumor stage

Characteristics	Hazard ratio (95% CI)	P value*
AJCC Tumor stage	2.775 (1.815-4.243)	< 0.001
Portal vein thrombosis	1.573 (0.912-2.715)	0.103
Serum AFP (≥ 400 ng/mL)	1.153 (0.688-1.919)	0.584
Serum PIVKA-II (≥ 300 mAU/mL)	2.718 (1.535-4.812)	0.001
Platelet count (≤ 130 × 10^3^/μL)	1.830 (1.130-2.964)	0.014

BCLC Tumor stage	1.210 (1.047-1.400)	0.010
Portal vein thrombosis	1.934 (1.094-3.422)	0.023
Serum AFP (≥ 400 ng/mL)	1.470 (0.888-2.433)	0.133
Serum PIVKA-II (≥ 300 mAU/mL)	3.688 (2.073-6.560)	< 0.001
Platelet count (≤ 130 × 10^3^/μL)	1.911 (1.187-3.076)	0.008

Abbreviation: AFP, alpha-fetoprotein; PIVKA-II, Protein induced by vitamin K absence or antagonist-II;

*P-values for each clinical variable calculated using Cox regression analysis.

### Subgroup analysis for extrahepatic metastases according to AJCC tumor stage

**Figure 2:**
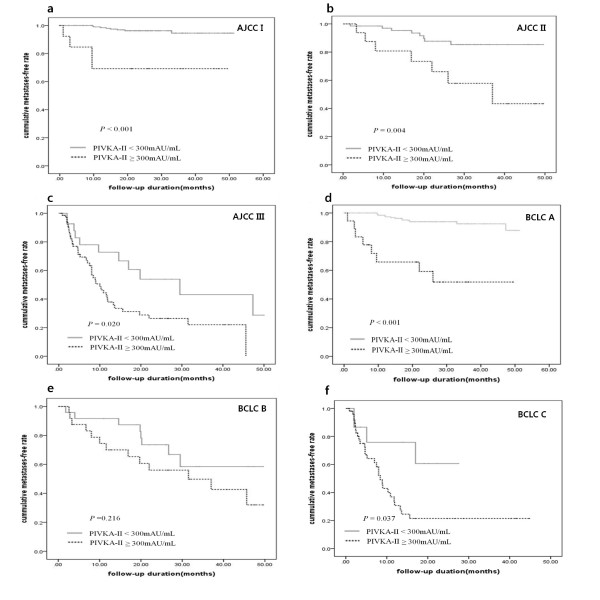
**The risks for extrahepatic metastases according to serum levels of PIVKA-II were assessed by the Kaplan-Meier method**. Risk of extrahepatic metastases was significantly higher for patients with a serum PIVKA-II level ≥ 300 mAU/mL than for those with PIVKA-II level < 300 mAU/mL among patients with stage I (a, P < 0.001), II (b, P = 0.004), and III (c, P = 0.020) HCC according to AJCC tumor stages and stage A (d, P < 0.001), B (e, P = 0.216) and C (f, P = 0.037) according to BCLC tumor stages.

To determine whether the serum levels of PIVKA-II predict HCC metastases in patients with different tumor stages, we stratified the patients according to tumor stage based on the AJCC staging system and evaluated the risk factors for extrahepatic metastases. When we analyzed the subgroups with all variables included at the time of diagnosis according to the AJCC tumor stage, the serum PIKVA-II (P < 0.001) and platelet count (P = 0.022) in stage I, serum AFP (P = 0.018) and PIVKA-II (P = 0.008) in stage II, and serum PIVKA-II (P = 0.023) and platelet count (P = 0.031) in stage III were risk factors for extrahepatic metastases in univariate analysis. In multivariate analysis, only the serum PIVKA-II level was an independent predictive factor of extrahepatic metastases in patients with stage I (P = 0.001), II (P = 0.023), and III (P = 0.028) disease. In particular, a serum PIVKA-II level ≥ 300 mAU/mL had a 8.8-fold (95% CI, 2.538-30.303, P < 0.001), 3.4-fold (95% CI, 1.179-9.523, P = 0.025), and 2.2-fold (95% CI, 1.086-4.504, P = 0.025) increased risk among patients with stages I, II, and III disease compared to patients with serum PIVKA-II levels < 300 mAU/mL in multivariate analysis after adjustment for AFP ≥ 400, PT, and platelet count. All other variables, including albumin, PT, AST, ALT, and bilirubin, were not associated with extrahepatic metastases in subgroup analysis according to stage. Table 4 shows the univariate and multivariate analysis for extrahepatic metastases with serum AFP, PIVKA-II, PT, and platelet count according to stage. The Kaplan curves for extrahepatic metastases according to AJCC stage are shown in Figure 2a (stage I, P < 0.001), 2b (stage II, P = 0.004), and 2c (stage III, P = 0.020). The median time to extrahepatic metastases in those with a PIVKA-II ≥ 300 mAU/mL was 37 months in stage II and 10 months in stage III, respectively, on Kaplan curves, whereas stage I patients with a PIVKA-II ≥ 300 mAU/mL did not reach the median time to extrahepatic metastases during follow up periods.

**Table 4:** Univariate and multivariate analysis according to stage

Characteristics	Hazard ratio (95% CI) in univariate analysis	P value*	Hazard ratio (95% CI) in multivariate analysis	P value*
**AJCC stage I**				

Serum AFP (≥ 400 ng/mL)	1.523 (0.412-5.629)	0.528	1.490 (0.394-5.649)	0.556
Serum PIVKA-II (≥ 300 mAU/mL)	9.317 (2.736-30.453)	< 0.001	8.771 (2.538-30.303)	0.001
PT (≤ 80%)	1.862 (0.503-6.896)	0.352	0.746 (0.164-3.397)	0.705
Platelet count (≤ 130 × 10^3^/μL)	4.608 (1.250-16.949)	0.022	4.948 (1.097-22.322)	0.037

**AJCC stage II**				

Serum AFP (≥ 400 ng/mL)	3.484 (1.233-9.803)	0.018	2.832 (0.983-8.196)	0.054
Serum PIVKA-II (≥ 300 mAU/mL)	3.984 (1.466-10.989)	0.008	3.355 (1.179-9.523)	0.023
PT (≤ 80%)	1.198 (0.434-3.305)	0.728	1.167 (0.382-3.564)	0.786
Platelet count (≤ 130 × 10^3^/μL)	1.164 (1.413-3.267)	0.773	0.820 (0.265-2.542)	0.732

**AJCC stage III**				

Serum AFP (≥ 400 ng/mL)	1.037 (0.586-1.834)	0.899	1, 164 (0.652-2.078)	0.608
Serum PIVKA-II (≥ 300 mAU/mL)	2.252 (1.116-4.545)	0.023	2.212 (1.086-4.504)	0.028
PT (≤ 80%)	1.192 (0.659-2, 158)	0.561	1.254 (0.685-2.296)	0.464
Platelet count (≤ 130 × 10^3^/μL)	1.934 (1.062-3.522)	0.031	1.868 (1.017-3.428)	0.044

**BCLC stage A**				

Serum AFP (≥ 400 ng/mL)	1.934 (1.094-3.424)	0.023	2.544 (1.003-6.451)	0.049
Serum PIVKA-II (≥ 300 mAU/mL)	9.319 (3.856-22.525)	< 0.001	8.403 (3.424-20.408)	< 0.001
PT (≤ 80%)	1.845 (0.714-4.761)	0.206	1.098 (0.404-2.9984)	0.854
Platelet count (≤ 130 × 10^3^/μL)	2.127 (0.896-5.050)	0.087	2.055 (0.820-5.153)	0.125

**BCLC stage B**				

Serum AFP (≥ 400 ng/mL)	1.183 (0.502-2.791)	0.701	1.163 (0.482-2.807)	0.737
Serum PIVKA-II (≥ 300 mAU/mL)	1.740 (0.715-4.232)	0.222	1.743 (0.711-4.271)	0.225
PT (≤ 80%)	1.054 (0.443-2.508)	0.905	1.142 (0.448-2.911)	0.782
Platelet count (≤ 130 × 10^3^/μL)	1.243 (0.461-2.710)	0.804	1.072 (0.424-2.712)	0.833

**BCLC stage C**				

Serum AFP (≥ 400 ng/mL)	1.382 (0.656-2.910)	0.394	1.377 (0.610-3.105)	0.440
Serum PIVKA-II (≥ 300 AU/mL)	2.909 (1.015-8.333)	0.047	3.174 (1.098-9.174)	0.033
PT (≤ 80%)	0.800 (0385-1.659)	0.548	0.588 (0.258-1.338)	0.205
Platelet count (≤ 130 × 10^3^/μL)	1.642 (0.790-3.412)	0.184	1.917 (0.893-4.114)	0.095

Abbreviation: AFP, alpha-fetoprotein; PIVKA-II, Protein induced by vitamin K absence or antagonist-II; PT, prothrombin time.

*P-values for each clinical variable calculated using Cox regression analysis.

### Subgroup analysis for extrahepatic metastases according to BCLC tumor stage

When we analyzed the subgroups with all variables included at the time of diagnosis according to the BCLC tumor stage, the serum PIKVA-II (P < 0.001) and serum AFP (P = 0.023) in stage A, and serum PIVKA-II (P = 0.047) in stage C were risk factors for extrahepatic metastases in univariate analysis. In multivariate analysis, the serum PIVKA-II level (P < 0.001) and AFP level (P = 0.049) in stage A and serum PIVKA-II (P = 0.033) in stage C were independent predictive factors of extrahepatic metastases. In BCLC staging B and D HCC patients, PIVKA-II was not a significant factor associated with extrahepatic metastases (P = 0.225, P = 0.678) in multivariate analysis. In particular, a serum PIVKA-II level ≥ 300 mAU/mL had a 8.4-fold (95% CI, 3.424-20.408, P < 0.001) and 3.2-fold (95% CI, 1.098-9.174, P = 0.033) increased risk among patients with stages A and C disease compared to patients with serum PIVKA-II levels < 300 mAU/mL. All other variables, including albumin, PT, platelet count, AST, ALT, and bilirubin, were not associated with extrahepatic metastases in subgroup analysis according to stage. The Kaplan curves for extrahepatic metastases according to BCLC stage are shown in Figure 2d (stage A, P < 0.001), 2e (stage B, P = 0.216), and 2f (stage C, P = 0.037). The median time to extrahepatic metastases in those with a PIVKA-II ≥ 300 mAU/mL was 31.5 in stage B and 8.4 months in stage C on Kaplan curves, whereas stage A patients with a PIVKA-II ≥ 300 mAU/mL did not reach the median time to extrahepatic metastases during follow up periods.

### Subgroup analysis according to treatment modality

The number of patients with extrahepatic metastasis was 59 (28.9%) for the TACE group, 7 (9.4%) for the PEIT group, 2 (4.7%) for the RFA group, and 8 (23.5%) for the surgery group. We analyzed the predictive factors for extrahepatic metastasis according to treatment modality. Serum PIVKA-II level (P < 0.001), AFP level (P = 0.027), portal vein thrombosis (P < 0.001), platelet count (P < 0.001), and PT (P = 0.018) were significantly associated with the presence of extrahepatic metastases in univariate analyses in the TACE group. It was consistently shown that PIVKA-II and platelet count were independent risk factors for extrahepatic metastases based on multivariate analysis. Patients with PIVKA-II ≥ 300 mAU/mL had a 6.4-fold increased risk (95% confidence interval [CI], 3.255-12.711, P < 0.001), and those with platelet count > 130 K had a 1.8-fold increased risk (95% confidence interval [CI], 1.084-3.154, P = 0.024) in the TACE group. AFP level and portal vein thrombosis did not appear statistically significant for extrahepatic metastasis in a multivariate analysis.

The serum PIVKA-II level (P = 0.007) and AFP level (P = 0.032) were significantly associated with the presence of extrahepatic metastases based on univariate analyses in patients receiving surgery. However, only the PIVKA-II level (P = 0.070) had an independent risk factor for extrahepatic metastases in multivariate analysis. A serum PIVKA-II level ≥ 300 mAU/mL had an 8.7-fold increased risk (95% CI, 4.793-15.699, P < 0.001) for extrahepatic metastasis. The serum PIVKA-II level (P = 0.012) and AFP level (P = 0.002) were significantly associated with the presence of extrahepatic metastases in patients receiving PEIT. A serum PIVKA-II level ≥ 300 mAU/mL had a 23.1-fold increased risk (95% CI, 2.175-245.979, P = 0.009) for extrahepatic metastasis, while a serum AFP level ≥ 400 mAU/mL had a 5.2-fold increased risk (95% CI, 1.000-27.742, P = 0.050). The serum PIVKA-II level (P = 0.019) was significantly associated with the presence of extrahepatic metastases in patients receiving RFA. However, no statistical significance manifested in patients with a serum PIVKA-II level ≥ 300 mAU/mL, which is due to the small number of patients.

### Subgroup analysis according to serum AFP and PIVKA-II levels

**Figure 3:**
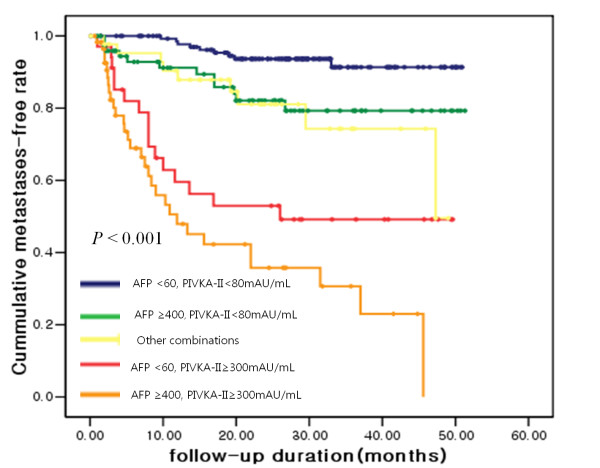
**The risk for extrahepatic metastases according to different combinations of serum AFP and PIVKA-II levels was assessed by the Kaplan-Meier method**.

To determine whether a specific combination of tumor markers is associated with the risk for metastases, we stratified the patients into 5 groups using the values of AFP and PIVKA-II at the time of diagnosis: group 1 patients were those who had a low AFP and a low PIVKA-II (AFP < 60 ng/mL and PIVKA-II < 80 mAU/mL); group 2 had a high AFP and a low PIVKA-II (AFP ≥ 400 ng/mL and PIVKA-II < 80 mAU/mL); group 4 had a low AFP and high PIVKA-II (AFP < 60 ng/mL and PIVKA-II ≥ 300 mAU/mL); group 5 had a high AFP and high PIVKA-II (AFP ≥ 400 ng/mL and PIVKA-II ≥ 300 mAU/mL); and group 3 included other combinations. The time to extrahepatic metastases based on the subgroup is presented using a Kaplan-Meier curve in Figure 3. When the relative risk was compared by Cox regression analysis, the patients in group 5 had a 9.7-fold relative risk for extrahepatic metastases compared to the patients in groups 1, 2, or 3 (95% CI, 5.780-16.393, P < 0.001), and the patients in group 4 had a 5.3-fold relative risk compared to the patients in groups 1, 2, or 3 (95% CI, 2.898-9.803, P < 0.001). Group 4 patients had a 2.7-fold relative risk for extrahepatic metastases compared to group 2 (95% CI, 1.886-6.134, P = 0.018); however, group 5 patients did not have an increased risk for extrahepatic metastases compared to group 4 (RR 1.8 fold, 95% CI, 0.959-3.278, P = 0.067). These results indicate that PIVKA-II levels are independently related to extrahepatic metastases regardless of AFP levels.

### Alteration of the PIVKA-II level during follow-up

**Figure 4:**
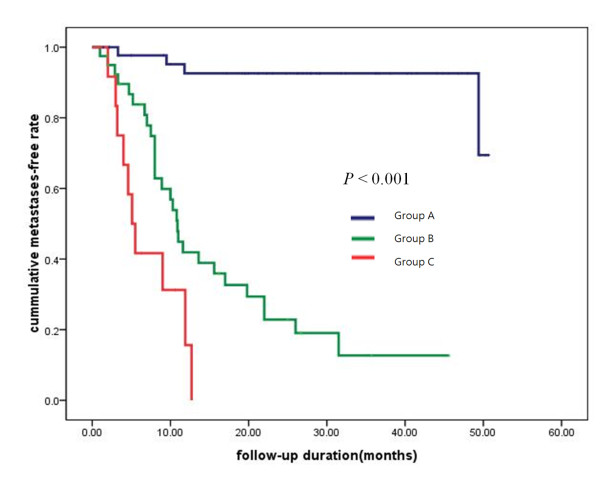
**The median time to extrahepatic metastases according to the change in the serum PIVKA-II level during follow-up and extrahepatic metastases in patients with elevated levels of PIVKA-II (P < 0.001)**.

We investigated the correlation between the change in the serum PIVKA-II level during follow-up and extrahepatic metastases in patients with elevated levels of PIVKA-II (**≥ **80 mAU/mL, 2.0 times the normal PIVKA-II level). One hundred thirty-three patients had serum levels of PIVKA-II ≥ 80 AU/mL at the time of diagnosis. Of these patients, 35 who did not have PIVKA-II level monitoring after initial management were excluded from this analysis. The remaining 98 patients were classified into the following 3 groups according to alterations in PIVKA-II levels during the follow-up periods: group A included patients in whom serum PIVKA-II values fell below the normal level after initial management and persisted below the normal level during all follow-up periods (n = 46); group B included patients in whom serum PIVKA-II levels fell below or above the normal range transiently compared to the initial value after treatment and then increased to **≥ **80 mAU/mL (n = 40); and group C included patients in whom serum PIVKA-II levels persistently increased above the initial value (n = 12) after initial treatment. All treatments were limited to reduce intrahepatic tumor burdens, such as TACE, RFA, and surgery. Median times to extrahepatic metastases in groups B and C were 10.9 and 5.1 months, respectively (P < 0.001, Figure 4); however, group C did not decrease to the median time to extrahepatic metastases during the follow-up period.

## Discussion

The results of this study have shown that the serum level of PIVKA-II is an independent prognostic factor for extrahepatic metastases in HCC patients. This association of PIVKA-II with extrahepatic metastases was maintained in the subgroup analyses, regardless of the tumor staging system used (AJCC and BCLC). Moreover, in the subgroup analysis involving the combination of AFP and PIVKA-II, group 4 (high PIVKA-II and low AFP) and 5 (high PIVKA-II and high AFP) showed an increased tendency for extrahepatic metastases and a shorter time to metastases than the other groups. Together, these findings suggest that the PIVKA-II levels are independently related to extrahepatic metastases regardless of serum AFP level and tumor staging; additionally, they showed a strong association with early stages with low AFP. This is consistent with prior clinical studies showing that the pre-operative PIVKA-II level is an independent predictor of microvessel invasion in post-surgical histology or after liver transplantation [15-17]. This finding may also be explained by an *in vitro *study showing that PIVKA-II stimulates cell proliferation and cell migration of vascular endothelial cells by binding to the kinase insert domain receptor, alternatively referred to as the vascular endothelial growth factor receptor-2 [18].

Several prior reports have suggested that PIVKA-II may be a useful marker for the prediction of portal vein thrombosis [19-22], as well as the intrahepatic spread of disease [22]. In addition, our study demonstrated that PIVKA-II levels may be strongly associated with extrahepatic metastases in patients with AJCC stage I and BCLC stage A who did not have portal vein thrombosis and invasion.

In the current study, PIVKA-II levels were strongly associated with extrahepatic metastases, especially in patients with early stage disease and smaller and/or fewer tumors compared to those in an advanced stage. These observations suggest that as the tumor stage advances, the association between PIVKA-II and the risk for metastases may be weakened. This can be explained by the intrahepatic tumor burden, which itself increases the value of PIVKA-II regardless of the presence of metastases. This can also be explained by follow-up data in patients with a high serum PIVKA-II level at the time of diagnosis. The patient group in which the serum PIVKA-II values decreased after intrahepatic tumor management and persisted below the normal range for PIVKA-II during the follow-up period could have only had an intrahepatic tumor before treatment. The high serum PIVKA-II level in this group reflects an intrahepatic tumor burden only. However, the patient group in which the serum PIVKA-II value transiently decreased after intrahepatic management and then increased after treatment could have had an intrahepatic tumor and undetected extrahepatic metastases before treatment. Thus, the value of PIVKA-II in this group increased by extrahepatic tumor growth during the follow-up period, but transiently decreased after intrahepatic tumor management. The last patient group in which the serum PIVKA-II value persistently increased, even after treatment, could have had an undetected higher extrahepatic tumor burden compared to the intrahepatic tumor; thus the value of PIVKA-II persistently increased, even if the intrahepatic tumors were controlled.

Our overall results may be explained by the different blood supply source to the tumor between intra- and extra-hepatic HCCs. Liver cells are mainly supplied by the portal vein, which conveys a sufficient amount of vitamin K directly by enterohepatic circulation from intestinal absorption by dietary intake or colonic microflora synthesis. Although intrahepatic HCCs are mainly supplied by the hepatic artery, intrahepatic HCCs are also supplied by the portal vein and are able to uptake a sufficient quantity of vitamin K. However, extrahepatic metastatic HCCs, such as those in lung and bone, receive blood from the peripheral circulation, which probably does not transport an adequate amount of vitamin K compared to the portal flow. Therefore, we can assume that HCC cells in extrahepatic metastases cannot actively synthesize prothrombin due to vitamin K insufficiency and may consequently increase the serum level of PIVKA-II. However, there are no studies which have compared the concentration of vitamin K between the portal vein and the peripheral blood or hepatic artery which support the portal vein having a higher concentration of vitamin K compared to the hepatic artery or systemic circulation.

In our study, we set the cutoff value for PIVKA-II levels at 300 mAU/mL and AFP level at 400 which was determined by the lowest value of ([1 - sensitivity]^2 ^+ [1 - specificity]^2^) as 349 mAU/mL and 420 ng/mL, respectively, by using a receiver operating characteristic curve of extrahepatic metastasis at 12 months after curative treatment. The analysis of gene networks associated with HCCs has shown that specific genotypes are associated with the biological characteristics of tumor development; for example, the HB subtype has been shown to be more invasive than the HC subtype [23]. Osteopontin is unique as a well-known gene associated with HCC metastases and progression [24]. There is no prior report implicating PIVKA-II as a predictive marker for extrahepatic metastases. Our study is the first prospective report to demonstrate an association between PIVKA-II levels and extrahepatic metastases in HCC patients. A corollary study is needed to determine whether a high PIVKA-II level is associated with a specific subtype of HCC.

## Conclusion

PIVKA-II levels might be a good candidate predictive marker for extrahepatic metastases, especially in patients with early HCC regardless of AFP.

## Competing interests

The authors declare that they have no competing interests.

## Authors' contributions

HMB conceived and designed the study, led the drafting of the manuscript, did the analysis and interpretation, and approved the final manuscript. JHL co-led the drafting of the manuscript, did interpretation, and approved the final manuscript. DSH and HSL provided financial and administrative support and approved the final manuscript. YJK participated in the interpretation of the results and approved the final manuscript. JHY, HSL, and YJK provided study material or patients. JHY designed the study, led the drafting of the manuscript, did the analysis and interpretation, and approved the final manuscript.

## Pre-publication history

The pre-publication history for this paper can be accessed here:

http://www.biomedcentral.com/1471-2407/11/435/prepub
